# Fatal Fulminant Hepatic Failure from Adenovirus in Allogeneic Bone Marrow Transplant Patients

**DOI:** 10.1155/2012/463569

**Published:** 2012-08-15

**Authors:** Jatin M. Vyas, Wayne A. Marasco

**Affiliations:** ^1^Division of Infectious Disease, Department of Medicine, Massachusetts General Hospital, Boston, MA 02114, USA; ^2^Department of Cancer Immunology and AIDS, Dana-Farber Cancer Institute, Brigham and Women's Hospital, Boston, MA 02215, USA

## Abstract

We report two cases of fatal hepatic failure in patients who received matched unrelated bone marrow transplantation. Both patients presented with high fevers, abnormal liver functions tests, and hypodense lesions in the liver by CT scan. Histologic examination of postmortem liver samples demonstrated extensive necrosis, and immunohistochemistry was positive for adenovirus.

## 1. Introduction

 Adenovirus infections have become more prevalent in immunocompromised patients [1, 2]. However, the lack of rapid diagnostic tests for adenovirus often delays prompt diagnosis that contributes to the morbidity and mortality in this patient population. We report two cases of fatal hepatic failure in patients who received matched unrelated bone marrow transplantation for hematologic malignancies. Adenovirus commonly causes respiratory, gastrointestinal, and genitourinary disease, but fulminant hepatic failure is rare [3]. Both patients presented with high fevers, abnormal LFTs, and hypodense lesions in the liver by computed tomography (CT) scan approximately 60–120 days after bone marrow transplantation. Despite maximum supportive care, both patients died despite in one case treatment with intravenous ribavirin. Histologic examination of post-mortem liver samples from both patients demonstrated extensive necrosis and immunohistochemistry was positive for adenovirus. Moreover, adenovirus was cultured from blood and liver. Adenovirus from these two patients was serotypes 2 and 5. Risk factors for the development of this type of infection include the presence of graft versus host disease and the use of immunosuppressive agents.

## 2. Cases

 Patient 1 was a 46-year-old male who initially presented with fatigue, headache, and easy bruising. Complete blood count (CBC) showed 221,000 white blood count (WBC) with 92% blasts, hematocrit (Hct) 22%, and platelets 46,000. He was diagnosed with acute lymphoblastic leukemia (ALL with t4:11). He was treated with hydrea and allopurinol, then daunorubicin, vincristine, cyclophosphamide, prednisone and intrathecal methotrexate. He developed fever and neutropenia and subsequent blood cultures grew *Streptococcus* group G, treated with vancomycin and ceftazidime and recovered. He underwent T cell-depleted allogeneic bone marrow transplantation 3 months later. His post-bone marrow transplantation course was complicated with fever and neutropenia, and blood cultures grew 1/2 bottles coagulase-negative *Staphylococcus* spp. He was treated with Vancomycin. He subsequently developed skin graft versus host disease (GVHD) that required treatment with 10 mg/kg methylprednisolone. Thirty days after bone marrow transplantation, he had worsening GVHD of skin, gut, and liver. He was treated with high-dose steroids, cyclosporine, octreotide and IL-2 receptor antibody (Zenapax). Forty-seven days after bone marrow transplantation, he developed fever to 38.9°C and a slight cough. He was treated with levofloxacin and symptoms resolved. Ten days later (57 days after bone marrow transplantation), he developed fever to 38.9°C, rigors, increasing lethargy, decreased appetite, and jaundice. He was restarted on levofloxacin and admitted to hospital. His past medical history was notable for being seropositive for cytomegalovirus (CMV) on ganciclovir. In addition to above medications, he was also maintained on atovaquone for *Pneumocystis jiroveci* pneumonia prophylaxis. He had three children at home (one child had “throat infection”) and no recent travel. He lived on Cape Cod, Massachusetts, USA. Prior to illness, he was a marathon runner.

**Figure 1:**
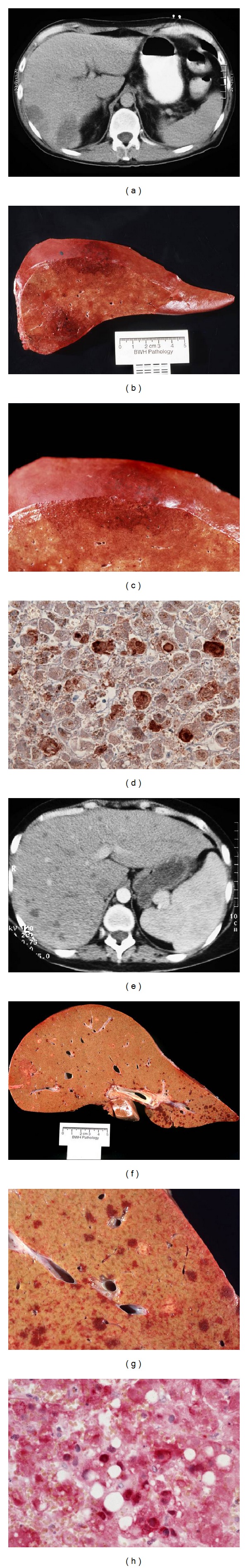
Radiographic and pathologic appearance of adenovirus infection of the liver. (Patient 1: Panels (a)–(d)) CT of the abdomen (a) showed two large hypodense lesions in the liver. On post-mortem examination, the liver had multiple large areas of necrosis ((b) and (c)). Immunohistochemistry of thin sections of the liver confirmed the presence of adenovirus (d). Subsequent testing revealed adenovirus, serotype 5. (Patient 2: Panels (e)–(h)) CT of the abdomen showed multiple small hypodense lesions in the liver (e). The liver had multiple small areas of necrosis ((f) and (g)). Immunohistochemistry of the liver confirmed the presence of adenovirus (h). Subsequent testing revealed adenovirus, serotype 2.

On admission to the hospital, he had a toxic appearance. He was somnolent, but arousable. His blood pressure was 100/60, heart rate was 120, respiratory rate was 24,f and temperature was 40.6°C. Notable findings on physical examination included icteric sclerae, left Hickman catheter in place, nontender, no erythema. His abdomen was soft, non-tender, nondistended, and no ascites on exam, and no hepatosplenomegaly. He had no skin lesions. Laboratory analysis revealed WBC 0.62, 65% polys, 5% bands, Hct 28.6%, Plts 29,000,f and abnormal liver function tests (Table 1). CT of the abdomen showed multiple large hypodense lesions in liver, but no splenic lesions (Figure 1(a)). Blood cultures revealed 2/4 bottles positive for coagulase negative *Staphylococcus* spp. His Hickman line was pulled. Serologies for viral hepatitis were negative, and CMV polymerase chain reaction (PCR) was negative from the blood. He developed hypotension requiring inotropic support and intubation for respiratory failure. A liver biopsy was not obtained secondary to patient's instability and bleeding diathesis. He developed acute renal failure requiring continuous veno-venous hemofiltration (CVVH). He developed disseminated intravascular coagulation (DIC) with continued bleeding and died on hospital day 5 despite maximum supportive care.

Patient 2 was a 38-year-old female with a history of Burkitt's lymphoma treated with McGrath protocol who underwent an allo-bone marrow transplantation. Her post bone marrow transplantation course was characterized by skin GVHD requiring high-dose steroids. She developed cough, low-grade fevers, and nasal stuffiness around 90 days post bone marrow transplantation. She started on clarithromycin for presumed bacterial sinusitis. She felt no better. She returned to clinic and was found to have new pancytopenia. Nasal washings were positive for parainfluenza type 1 and admitted to hospital for possible trial of ribavirin. Physical examination revealed mild frontal sinus tenderness. Initial laboratories showed WBC 0.9 with 62% PMNs, Hct 24.9%, and Plts of 18,000. LFTs were abnormal (Table 1).

Within 48 hours of hospital admission, she developed fever and neutropenia. Levofloxacin and gentamicin were administered. Chest X-ray and chest CT showed no parenchymal abnormalities, though upper cuts of liver showed new lesions. A dedicated abdominal CT showed multiple small hypodense lesions in the liver, spleen, and a large dilated transverse colon (Figure 1(e)). She developed bloody diarrhea, and found to be *C. difficile* toxin positive. Oral metronidazole was initiated. She was also started on high-dose ambisome for presumed hepatosplenic candidiasis based on-the-CT findings. Serologies were negative for hepatitis B and C. There was no evidence of CMV replication by PCR from the blood. Bone marrow was negative for the presence of HHV-6. On day 119 post-bone marrow transplantation (hospital day number 7), she developed hypotension requiring pressors, and was intubated. Her antimicrobials were broadened to vancomycin, ceftazidime, gentamicin, metronidazole, and acyclovir. Liver biopsy was not obtained secondary to hemodynamic instability and bleeding diathesis. Despite maximal support and three doses of IV ribavirin, she died four days later. Adenovirus serotype 2 was cultured from blood, liver tissue, and bone marrow.

## 3. Discussion

 Adenovirus commonly causes pulmonary, gastrointestinal, and genitourinary disease in immunocompromised patients, but fulminant hepatic failure is rare [4]. The incidence of adenoviral infection in the transplant population ranges between 3% and 29% in HSCT recipients, 5 and 10% in solid organ recipients [5]. Symptoms at presentation predominately depended on the site of infection (e.g., hematuria for cystitis, diarrhea for colitis). In contrast, these two patients had nonspecific symptoms and then developed non-specific abdominal pain late in the presentation of this disease. The clinical features of fatal hepatic necrosis caused by adenovirus include high fevers, rapid rise in transaminases, a spectrum of radiographic abnormalities in the liver by CT, hemodynamic lability, and a bleeding diathesis. It is interesting to note that bone marrow suppression may be an early indicator of disseminated adenovirus infections as seen in these two patients.

While both patients shared the diagnosis of fatal hepatic failure secondary to adenovirus infection, the radiographic appearance of the hepatic abnormalities showed striking differences. In the first case, the abdominal CT scan demonstrated two large hypodense lesions in the liver. The remainder of the liver appeared normal radiographically. Upon visual inspection of the liver, there were two discrete areas of necrosis with the remainder of the tissue being normal (Figures 1(b) and 1(c)). Immunohistologic examination of thin sections of the liver parenchyma confirmed the presence of adenovirus (Figure 1(d)). Subsequent testing revealed that the adenovirus serotype 5 was present (data not shown). In sharp contrast, the abdominal CT imaging in the second case showed multiple small hypodense lesions with no single lesion predominating. Post-mortem examination of the organ showed small lakes of necrosis (Figures 1(f) and 1(g)). Finally, immunohistologic staining revealed the presence of adenovirus (Figure 1(h)), serotype 2. Previous reports have not supported the assertion that different serotypes present with distinct liver parenchymal abnormalities [6–13]. While both patients were immunocompromised, it is likely that the degree and magnitude of their immunosuppression contributed to their clinical presentation [14]. 

T cell-depleted bone marrow transplantation has been linked to prolonged immunologic recovery and increased risk of infections [14]. While serious CMV infection has been documented in patients who have received this type of bone marrow transplantation [15], disseminated adenoviral infections have been rarely reported [16]. Innate immunity to adenovirus infections involves the TLR2 and TLR9 pathway [17], and emerging evidence indicates that the inflammasome participates in the antiviral response [18]. Additionally, adaptive immunity using CD4^+^ and CD8^+^ antigen-specific T cells are required for complete and persistent antiviral activity [17]. These observations suggest that specific therapies that augment these responses including Toll-like receptor (TLR) agonists, or adoptive transfer to antigen-specific T cells may represent promising avenues of new treatment in these infected patients. Newer methods of detection of adenovirus DNA in the blood by quantitative PCR are being utilized for the evaluation of adenovirus infections in immunocompromised patients. Rising levels of adenovirus DNA present in the blood in the correct clinical context may provide a clinical diagnosis, but this test was not readily available when these cases presented. Additionally, emerging modalities of gene replacement that rely on the delivery of exogenous genes to patients using adenovirus should be used with caution in these immunocompromised patients. Knowledge of the immune defects seen in bone marrow transplant patients is necessary to provide guidance of the safety and utility of adenoviral viral vectors. Clinicians who care for bone marrow transplant patients should be aware of the possibility of adenovirus infection in patients presenting with fulminant hepatic failure.

## Figures and Tables

**Table 1a:** (a) Days after BMtx

Patient 1	57	58	59	60	61
ALT	450	1015	5830	9150	4070
AST	424	1121	8250	16050	7970
LDH			9870		10140
Alk Phos	254	190	173		265
T Bil	2.1	4	4.5	7	6.3
D Bil	1.2		2.4		2.5
Alb	2.4	2.5	2.9		

**Table 1b:** (b) Days after BMtx

Patient 2	109	113	114	115	116	117	118	119	120	121	122	123
ALT												1500
AST	58	164	131	149	277	469	605	1027	2030	2725	4440	6720
LDH	956	1393								4810	8130	
Alk Phos	174	473	302	281	411	540	549	557	638	564	419	711
T Bil	1.2	1.3	2.5	2.3	2.5	4.5	5.1	5.9	6.4	6.5	5	4.8
D Bil	0.7	1	1.2			2.9					3	2.9
